# Cognitive Impairment and Dementia in Primary Care: Current Knowledge and Future Directions Based on Findings From a Large Cross-Sectional Study in Crete, Greece

**DOI:** 10.3389/fmed.2020.592924

**Published:** 2020-11-23

**Authors:** Antonios Bertsias, Emmanouil Symvoulakis, Chariklia Tziraki, Symeon Panagiotakis, Lambros Mathioudakis, Ioannis Zaganas, Maria Basta, Dimitrios Boumpas, Panagiotis Simos, Alexandros Vgontzas, Christos Lionis

**Affiliations:** ^1^Clinic of Social and Family Medicine, School of Medicine, University of Crete, Heraklion, Greece; ^2^MELABEV - Community Clubs for Eldercare, Research and Development Department, Jerusalem, Israel; ^3^Department of Internal Medicine, School of Medicine, University of Crete, Heraklion, Greece; ^4^Department of Neurology, School of Medicine, University of Crete, Heraklion, Greece; ^5^Department of Psychiatry, School of Medicine, University of Crete, Heraklion, Greece; ^6^Department of Internal Medicine, School of Medicine, University of Athens, Athens, Greece; ^7^Computational Biomedicine Lab, Institute of Computer Science, Foundation for Research and Technology-Hellas, Herakleion, Greece

**Keywords:** dementia, Alzheimer's disease, mild cognitive impairment (MCI), primary health care (PHC), neurocognitive impairment

## Abstract

**Introduction:** Dementia severely affects the quality of life of patients and their caregivers; however, it is often not adequately addressed in the context of a primary care consultation, especially in patients with multi-morbidity.

**Study Population and Methods:** A cross-sectional study was conducted between March-2013 and December-2014 among 3,140 consecutive patients aged >60 years visiting 14 primary health care practices in Crete, Greece. The Mini-Mental-State-Examination [MMSE] was used to measure cognitive status using the conventional 24-point cut-off. Participants who scored low on MMSE were matched with a group of elders scoring >24 points, according to age and education; both groups underwent comprehensive neuropsychiatric and neuropsychological assessment. For the diagnosis of dementia and Mild-Cognitive-Impairment (MCI), the Diagnostic and Statistical Manual-of-Mental-Disorders (DSM-IV) criteria and the International-Working-Group (IWG) criteria were used. Chronic conditions were categorized according to ICD-10 categories. Logistic regression was used to provide associations between chronic illnesses and cognitive impairment according to MMSE scores. Generalized Linear Model Lasso Regularization was used for feature selection in MMSE items. A two-layer artificial neural network model was used to classify participants as impaired (dementia/MCI) vs. non-impaired.

**Results:** In the total sample of 3,140 participants (42.1% men; mean age 73.7 SD = 7.8 years), low MMSE scores were identified in 645 (20.5%) participants. Among participants with low MMSE scores 344 (54.1%) underwent comprehensive neuropsychiatric evaluation and 185 (53.8%) were diagnosed with Mild-Cognitive-Impairment (MCI) and 118 (34.3%) with dementia. Mental and behavioral disorders (F00-F99) and diseases of the nervous system (G00-G99) increased the odds of low MMSE scores in both genders. Generalized linear model lasso regularization indicated that 7/30 MMSE questions contributed the most to the classification of patients as impaired (dementia/MCI) vs. non-impaired with a combined accuracy of 82.0%. These MMSE items were questions 5, 13, 19, 20, 22, 23, and 26 of the Greek version of MMSE assessing orientation in time, repetition, calculation, registration, and visuo-constructive ability.

**Conclusions:** Our study identified certain chronic illness-complexes that were associated with low MMSE scores within the context of primary care consultation. Also, our analysis indicated that seven MMSE items provide strong evidence for the presence of dementia or MCI.

## Introduction

Dementia [or major neurocognitive impairment (1)] is characterized by significant decline from a previously attained level of cognitive and everyday functioning (2). Among chronic conditions dementia is one of the greatest global challenges for health and social care in the 21st century with over 50 million sufferers worldwide, expected to show a 3-fold increase by the year 2050 (3). Since there is no treatment available to stop or reverse the underlying pathophysiology, the economic, and societal repercussions of dementia threaten to become overwhelming as more people live into old age (4). Decline in one or more cognitive domains which is not sufficiently severe to significantly impair everyday functioning is rapidly recognized as a chronic condition that affects quality of life among elders. This condition which is collectively referred to as Mild Cognitive Impairment [MCI or minor neurocognitive impairment (1)]. MCI is further important as it is associated with higher likelihood of emergence of dementia than average (5).

General practitioners and family physicians (GPs/FPs) are typically the first point of contact with the health-care system so they are ideally positioned in order to provide care for individuals living with dementia from early to end stages of the illness (6). Primary care professionals including GPs/FPs share a full and long-term understanding of the medical, social, and mental health situations of these patients and their families (6). Despite that, the diagnosis of dementia can be delayed by the insidiousness of the symptoms and the perceptions by both patients and GPs/FPs that it may be just a sign of normal aging (7). As many as two-thirds of people with dementia may be misdiagnosed and there is often a significant delay between symptom onset and diagnosis (8). Even in high-income countries with advanced health-care systems, more than half of dementia cases are not formally diagnosed (9–11). A recent systematic review identified several system-related factors contributing to missed or delayed diagnosis, including limited time with patients, few specialists available for consultation and limitations on diagnostic tests recommended by management guidelines (12).

To this end, a growing number of studies have focused on developing algorithms to facilitate early identification of age-related cognitive disorders based on information readily available at the primary care level (13, 14). These efforts have been enhanced with the introduction of machine learning algorithms in medicine (15, 16). A recent review indicated that Deep learning approaches, such as convolutional neural networks (CNN) or recurrent neural networks (RNN), that use neuroimaging data without pre-processing for feature selection have yielded accuracies of up to 96.0% for AD classification and 84.2% for prediction of conversion to dementia from MCI with the best classification performance achieved when multimodal neuroimaging and cerebrospinal fluid biomarkers were combined (17). Machine learning algorithms have also been used to identify dementia from administrative claims dataset (18) or even identify the importance of related risk-factors (19).

In Greece until recently there was a paucity of data regarding dementia and cognitive impairment in general. Most data were from small-scale studies in selected populations (20, 21). Some recent studies provided new insights regarding the prevalence and geo-epidemiology of dementia and MCI in the country (22–26). As part of this effort, a multi-disciplinary research network for the study of dementia was established within the Faculty of Medicine at the University of Crete, Greece. This network includes scientists and practitioners from various medical disciplines including General Practitioners (GPs) and nurses serving in the community and secondary health care specialists (geriatricians, neurologists, neuropsychologists, and psychiatrists). The overall goal of the project was to create a clinical and research network of excellence within the University of Crete, to develop diagnostic tools for the detection of age-related cognitive decline, including Alzheimer's disease, and to identify epidemiologic and genetic determinants for development and progress of the disease (22).

This study utilizes data from the Cretan Aging Cohort (22) in order to provide new insights regarding dementia in Primary Care. The overall aim is to provide information about cognitive impairment attributed to dementia or MCI which will assist GPs/FPs and primary care professionals in identifying dementia or MCI taking into account the potentially limited time and resources. The research questions that this study seeks to address are:

1. Are chronic-illness complexes, as expressed by ICD-10 categories, associated with cognitive impairment attributed to dementia or MCI in elder individuals?
2. Is there a relationship between multi-morbidity and cognitive impairment?
3. Are any chronic illnesses more prevalent in patients suffering from MCI, dementia compared to non-impaired elder individuals?
4. Can we generate a brief cognitive test based on MMSE items using machine learning algorithms?

## Methods

### Study Design

This study utilizes data from the Cretan Aging Cohort [CAC] (22). The CAC is a study of 3,140 community-dwelling individuals aged 60 years or older that took place between May 2014 and December 2015 at the District of Heraklion in the island of Crete, Greece. The CAC was a two-phase project involving both a PHC team as well as a secondary health care team, in terms of a mutual contribution to a multidisciplinary collaboration.

### Participants

Eligible participants were consecutive visitors of a well-defined PHC setting. Eligible PHC units were staffed by GPs who were members of an established PHC research network which is coordinated by the Clinic of Social and Family Medicine, Faculty of Medicine, University of Crete, Greece. A total of 14 PHC units participated in the study. Eleven of the participating PHC units were located in rural or semi-urban locations, serving a population of 100,800 individuals and all were public (the total eligible public PHC units in rural/semi-urban areas were 21). Three of participating PHC units were located in the city of Heraklion serving a population of 204,690 individuals, one was public (the only public PHC unit in the city of Heraklion at that time) and two were private.

Eligible participants were those aged 60 years or older. All PHC visitors who were acutely ill or demanded urgent care or hospitalization were excluded from the invitation process. Established diagnosis of dementia or MCI was not established as an exclusion factor. Eligible participants were invited by the trained GPs to participate in the study. The interviews of the initial sample of 3,140 individuals were conducted by trained nurses supervised by participating GPs. Participant's companions were asked to provide information in cases where participants had difficulty/could not recall and provide with adequate information. Participant responses on clinically-relevant questions were later verified. Upon interview completion, by their GPs performed a cross-check of the questionnaire. Evaluation of cognitive function was conducted using the Greek version of the Mini Mental State Examination (MMSE) test (27, 28).

Participants showing cognitive impairment according to their MMSE scores (*n* = 636) were then invited for a thorough neuropsychiatric and neuropsychological assessment (Phase II) by a group of secondary health care experts. 344 out of the 636 (54.1%) invited participants accepted participation. Those who denied participation (*n* = 292; refused, could not be located, or passed away) did not differ from the 636 subjects referred, in terms of age, sex, and BMI. A matched sample of 181 individuals (matched for place of residency) with MMSE>24 (*n* = 2,504) were also invited for a thorough assessment and 161/181 accepted participation resulting in a total of 505 individuals that participated in Phase II of the study. A semi-structured interview with an approximate duration of 2 h was conducted either at the University Hospital of Heraklion, either at the local Health Centers or at participants' homes if more convenient for some participants. Certified psychiatrists, neurologists, gerontologists, and health-care research assistants conducted the assessments.

### Measurements

A structured and pre-tested questionnaire was used in order to collect the requested information from participants and their caregivers. This questionnaire included socio-demographic information (date of birth, gender, place of residency, marital status, level and years of formal education, living situation, and current/former employment status), health/life-style habits (smoking and alcohol consumption) and anthropometric information (height, weight, waist circumference). Chronic conditions were self-reported by patients or by their caregivers and cross-validated by their GPs using the patient's electronic health record. From the list of chronic illnesses the Charlson co-morbidity index was calculated (29). All chronic illnesses were then categorized in ICD-10 categories (30). Finally, the questionnaire included the Greek version of the MMSE (27, 28) in order to assess general cognitive ability using a universal cut-off of 23/24 points. MMSE was chosen as a screening tool since at the time of the study it was the only translated and validated instrument that could be used in the context of Greek primary care. Participants with MMSE scores <24 units were referred for further assessment (Phase II) described above. For the diagnosis of dementia and MCI, the Diagnostic and Statistical Manual of Mental Disorders, Fourth ed. criteria and the International Working Group criteria were used, respectively (31).

### Statistical Analysis

Demographic and other characteristics were summarized using descriptive statistics. Between-gender univariate comparisons were made using Pearson's chi-square test of independence (for categorical variables) and independent samples *t*-test (for continuous variables).

Multiple logistic regression models stratified by gender and adjusted for age and level of education were used in to assess possible associations between chronic illnesses in ICD-10 categories (yes/no) and low MMSE scores (yes/no). The ICD-10 categories included in the analysis were (1) Diseases of the blood and blood-forming organs and certain disorders involving the immune mechanism (D50-D89), (2) Endocrine, nutritional and metabolic diseases (E00-E90), Mental and behavioral disorders (F00-F99), (3) Diseases of the nervous system [excluding dementia or related conditions] (G00-G99), (4) Diseases of the eye and adnexa (H00-H59), (5) Diseases of the ear and mastoid process (H60-H95), (6) Diseases of the circulatory system (I00-I99), (7) Diseases of the respiratory system (J00-J99), (8) Diseases of the digestive system (K00-K93), (9) Diseases of the musculoskeletal system and connective tissue (M00-M99), and (10) Injury, poisoning, and certain other consequences of external causes (S00-T98).

Generalized linear LASSO (Least Absolute Shrinkage and Selection Operator) regularization was used to identify the most important predictors among the 30 MMSE items in the subsample of participants with an established formal diagnosis from Phase II of the project. Formal diagnosis, i.e., cognitively non-impaired vs. cognitively impaired (dementia and MCI grouped together) served as the dependent variable. Diagnostic accuracy of selected items was assessed using a two-layer artificial neural networks model. Six-fold cross-validation was conducted (on each fold the sample was randomly split to 70% training, 15% testing, 15% validation). The level of significance was set to 5%, IBM SPSS 24 and Python scikit-learn (version 0.23.1) were used to conduct analyses.

## Results

### Correlates of Low MMSE Scores

Three thousand four hundred seventy-one individuals, visitors of the selected PHC units were invited to participate. Two hundred and seventy-one (7.8%) declined participation. The reasons for non-participation were lack of time for the interview (*n* = 217; 80%) and unwillingness to participate in research (*n* = 54; 20%). Database was checked for duplicate entries, data consistency, and missing values and a total of 3,140 individuals were included in the final analysis.

### Sociodemographic, Somatometric, and Life-Style Characteristics

The majority of participants were women (*n* = 1,785; 56.8%). Most participants (*n* = 1,303; 41.5%) were 70–79 years old and 777 (24.7%) were 80 years old or older. Women were significantly younger compared to men with 650 (35.8%) being 60–69 years old compared to 410 (31.0%) men. On the contrary, more men were aged 80 years or older compared to women (*n* = 384; 29.9 vs. 393; 21.6%; *p* < 0.0001). The majority of participants were married, yet women were widowed to a significantly higher extent compared to men (*n* = 628; 34.7% vs. *n* = 137; 10.4%; *p* < 0.0001). Parallel to this 695 (22.3%) reported living alone, with this rate being significantly higher amongst women compared to men (*n* = 557; 30.9% vs. *n* = 138; 10.5%; *p* < 0.0001). Most participants reported having received primary education (*n* = 2,305; 73.7%), while 253 (8.1%) reported not having received any formal education. Education level was significantly higher in men compared to women with men having received secondary or tertiary level of education to a significantly higher extent compared to women (*p* < 0.0001).

As shown in Table 1 almost half of the participants were obese (*n* = 1,396; 45.4%) with women being obese at a significantly higher extent compared to men (*n* = 928; 52.3% vs. 468; 35.9%; *p* < 0.0001). About one third of participants reported being ever smoker (current or former) with the extent of smoking being much more prevalent in men compared to women (*n* = 914; 69.3% vs. *n* = 251; 13.9%; *p* < 0.0001). Finally, half participants (*n* = 1,636; 52.3%) reported being ever alcohol users (current or former), with alcohol usage being more prevalent in men compared to women (*n* = 1,058; 80.3% vs. *n* = 578; 31.9%; *p* < 0.0001).

**Table 1:** Demographic, somatometric, and life-style characteristics of participants and between-gender comparisons.

	**Overall** **(*n* = 3,140)**	**Women** **(*n* = 1,785)**	**Men** **(*n* = 1,355)**	***P*-value**
**Age**, mean (SD)	73.8 (7.8)	73.1 (7.6)	74.5 (7.9)	<0.0001
**Age groups**				<0.0001
(60–69) years	1,060 (33.8%)	650 (35.8%)	410 (31.0%)	
(70–79) years	1,303 (41.5%)	774 (42.6%)	529 (40.0%)	
80+ years	777 (24.7%)	393 (21.6%)	384 (29.9%)	
**Marital status^a^**				<0.0001
Single	88 (2.8%)	56 (3.1%)	32 (2.4%)	
Married	2,216 (70.8%)	1,096 (60.5%)	1,120 (84.8%)	
Divorced	63 (2.0%)	32 (1.8%)	31 (2.3%)	
Widowed	765 (24.4%)	628 (34.7%)	137 (10.4%)	
**Lives alone (yes)^b^**				<0.0001
	695 (22.3%)	557 (30.9%)	138 (10.5%)	
**Level of education^c^**				<0.0001
None	253 (8.1%)	184 (10.2%)	69 (5.2%)	
Primary	2,305 (73.7%)	1,365 (75.5%)	940 (71.2%)	
Secondary	425 (13.6%)	207 (11.5%)	218 (16.5%)	
University/college	145 (4.7%)	51 (2.8%)	94 (7.1%)	
**Obese (yes)^d^**	1,396 (45.4%)	928 (52.3%)	468 (35.9%)	<0.0001
**Ever smoker (yes)^e^**	1,165 (37.3%)	251 (13.9%)	914 (69.3%)	<0.0001
**Ever alcohol user (yes)^f^**	1,636 (52.3%)	578 (31.9%)	1,058 (80.3%)	<0.0001

a *8 missing values*,

b *28 missing values*,

c *12 missing values*,

d *62 missing values*,

e *13 missing values*,

f *10 missing values*.

### Chronic Illness Complexes and Multi-Morbidity: Between Gender Comparisons

The most prevalent chronic illnesses and conditions were endocrine, nutritional, and metabolic diseases (E00-E99) with 2,400 (78.0%) reported suffering from at least one such condition, followed by the diseases of the circulatory system (I00-I99) (*n* = 2,253; 71.8%), diseases of the musculoskeletal system and connective tissue (M00-M99) (*n* = 840; 26.8%), diseases of the digestive system (K00-K93) (*n* = 832; 26.5%), mental and behavioral disorders (F00-F99) (*n* = 523; 16.7%) and diseases of the respiratory system (J00-J99) (*n* = 347; 11.1%; see Table 2). The rates of chronic illnesses grouped in ICD-10 categories were significantly different between the two genders, being more prevalent in women compared to men, with the exemption of the diseases of the eye and adnexa (H00-H99) that were significantly more prevalent in men compared to women (*n* = 120; 8.5% vs. *n* = 109; 6.0%; 0 = 0.008). Participants assessed their health status at an average of 6.9/10 points (SD = 2.2), with higher rates by men than women (7.4 ± 2.1 vs. 6.6 ± 2.3; *p* < 0.0001). This was consistent with a greater average number of chronic illnesses in women as compared to men (3.5 ± 1.9 vs. 3.1 ± 1.8; *p* < 0.0001). On the other hand, the mean Charlson index scores were significantly higher in men compared to women (4.1 ± 1.0 vs. 4.1 ± 0.9; *p* < 0.0001). Low MMSE scores were detected in one out of five participants (*n* = 645; 20.6%) with the prevalence of low MMSE scores being almost double in women compared to men (*n* = 469; 26.0% vs. *n* = 176; 13.3%; *p* < 0.0001).

**Table 2:** Frequency of chronic illnesses by ICD-10 category, stratified by gender.

**ICD-10 category (yes/no)**	**Overall**	**Women** **(*n*, %)**	**Men** **(*n*, %)**	***P*-value**
Endocrine, nutritional, and metabolic diseases (E00-E90)	2,400 (78.0%)	1,474 (83.1%)	926 (71.1%)	<0.0001
Diseases of the circulatory system (I00-I99)	2,253 (71.8%)	1,311 (72.2%)	942 (71.3%)	0.549
Diseases of the musculoskeletal system and connective tissue (M00-M99)	840 (26.8%)	733 (40.4%)	107 (8.1%)	<0.0001
Diseases of the digestive system (K00-K93)	832 (26.5%)	510 (28.1%)	322 (24.4%)	0.020
Mental and behavioral disorders (F00-F99)	523 (16.7%)	370 (20.4%)	153 (11.6%)	<0.0001
Diseases of the respiratory system (J00-J99)	347 (11.1%)	142 (7.8%)	205 (15.5%)	<0.0001
Diseases of the ear and mastoid process (H60-H95)	317 (10.1%)	219 (12.1%)	98 (7.4%)	<0.0001
Diseases of the eye and adnexa (H00-H59)	221 (7.0%)	109 (6.0%)	120 (8.5%)	0.008
Diseases of the nervous system (G00-G99)	181 (5.8%)	103 (5.7%)	78 (5.9%)	0.789
Diseases of the blood and blood-forming organs and certain disorders involving the immune mechanism (D50-D89)	179 (5.7%)	112 (6.2%)	67 (5.1%)	0.188
Injury, poisoning, and certain other consequences of external causes (S00-T98)	108 (3.4%)	67 (3.7%)	41 (3.1%)	0.372
Health status self-assessment mean (SD)	6.9 (2.2)	6.6 (2.3)	7.4 (2.1)	<0.0001
Number of chronic conditions Mean (SD)	3.3 (1.8)	3.5 (1.9)	3.1 (1.8)	<0.0001
Charlson index score, mean (SD)	4.2 (1.0)	4.1 (0.9)	4.3 (1.0)	<0.0001
Low MMSE (yes)	645 (20.6%)	469 (26.0%)	176 (13.3%)	<0.0001

### Chronic Illness Complexes and Low MMSE Scores: Multivariate Analyses

Table 3 presents the adjusted odds ratios of having a low MMSE score by ICD-10 category. Significant associations between presence of Mental and behavioral disorders (F00-F99) were identified in both genders (Odds Ratio [OR] 1.71; 95% CI from 1.29 to 2.25 in women and OR 2.27; 95% CI from 1.46 to 3.56 in men). Furthermore, the odds of low MMSE-score were higher among participants with a history of diseases of the central nervous system (G00-G99) in both genders (OR 1.65; 95% CI 1.04–2.59 in women and OR 1.82; 95% CI from 1.02 to 3.29 in men; *p* < 0.0001). Low MMSE score was further associated with history of injury, poisoning, and other consequences of external causes (S00-T98) in men only (OR 2.99; 95% CI from 1.39 to 6.43; *p* = 0.005). Finally, an inverse relationship between the diseases of the musculoskeletal system and connective tissue (M00-M99) and low MMSE scores was identified only in women (OR 0.77; 95% CI 0.61–0.99; *p* = 0.042). The number of chronic illnesses did not increase the odds of having low MMSE scores in neither gender whilst the Charlson index score was associated with increased odds of low MMSE scores men (OR 1.31; 95% CI 1.09–1.57; *p* = 0.004).

**Table 3:** Logistic regression models predicting odds of having low MMSE-score by ICD-10 category, stratified by gender, and adjusted for age and years of formal education.

**ICD-10 category (yes/no)**	**Women** **(*n* = 1,785)** **Adjusted OR**	**95% CI** **(*p*-value)**	**Men** **(*n* = 1,355)** **Adjusted OR**	**95% CI** **(*p*-value)**
Diseases of the blood and blood-forming organs and certain disorders involving the immune mechanism (D50-D89)	1.16 (0.73–1.84)	0.524	0.59 (0.25–1.38)	0.226
Endocrine, nutritional and metabolic diseases (E00-E90)	0.83 (0.61–1.14)	0.254	0.59 (0.62–1.31)	0.591
Mental and behavioral disorders (F00-F99)	1.71 (1.29–2.25)	<0.0001	2.27 (1.46–3.56)	<0.0001
Diseases of the nervous system (G00-G99)	1.65 (1.04–2.59)	0.032	1.82 (1.02–3.29)	0.046
Diseases of the eye and adnexa (H00-H59)	1.31 (0.83–2.08)	0.246	0.77 (0.43–1.38)	0.375
Diseases of the ear and mastoid process (H60-H95)	0.76 (0.54–1.09)	0.134	0.60 (0.32–1.16)	0.127
Diseases of the circulatory system (I00-I99)	0.93 (0.71–1.24)	0.628	0.88 (0.69–1.53)	0.883
Diseases of the respiratory system (J00-J99)	0.99 (0.64–1.53)	0.971	0.80 (0.51–1.26)	0.336
Diseases of the digestive system (K00-K93)	1.18 (0.91–1.53)	0.203	0.96 (0.69–1.49)	0.956
Diseases of the musculoskeletal system and connective tissue (M00-M99)	0.77 (0.61–0.99)	0.042	0.71 (0.39–1.31)	0.271
Injury, poisoning, and certain other consequences of external causes (S00-T98)	1.25 (0.68–2.31)	0.479	2.99 (1.39–6.43)	0.005
Number of chronic conditions	0.99 (0.93–1.06)	0.872	1.02 (0.93–1.12)	0.729
Charlson index score	1.14 (0.98–1.32)	0.082	1.31 (1.09–1.57)	0.004

### Frequency of Specific Chronic Illnesses in Participants Diagnosed With Dementia or MCI

In Table 4 the frequencies of chronic illnesses of participants diagnosed with dementia, MCI, and non-impaired participants are presented. Significant differences were identified in the frequency of CHD with the prevalence being higher in participants diagnosed with dementia (*n* = 30; 23.8%) compared to non-impaired participants (*n* = 22; 15.1%) and participants with MCI (*n* = 27; 11.7%; *p* = 0.011). Similar patterns were identified in the prevalence of depression with the rates being higher in participants diagnosed with dementia (*n* = 37; 29.4%) vs. non-impaired (*n* = 19; 13.1%) and those with MCI (*n* = 39; 16.9%). On the contrary, the frequency of osteoporosis was higher amongst participants with MCI (*n* = 69; 30.0%) vs. non-impaired (*n* = 31; 21.4%) and participants with dementia (*n* = 22; 17.5%); *p* = 0.019. A similar pattern was found for arthritis, with higher rates in the MCI (*n* = 37; 16.0%) as compared to the non-impaired (*n* = 14; 9.6%) and dementia groups (*n* = 11; 8.7%) with the results being close to statistical significance (*p* = 0.066). Finally, GERD was more frequent in participants with MCI (*n* = 57; 24.7%) as compared to non-impaired (*n* = 24; 16.4%) and participants with dementia (*n* = 21; 16.7%; *p* = 0.078). The mean number of chronic illnesses and the Charlson index was significantly higher among participants with dementia (4.1 ± 1.9 and 4.9 ± 0.9, respectively) as compared to non-impaired (3.5 ± 2.1 and 4.2 ± 0.8, respectively) and MCI groups (3.6 ± 1.9 and 4.4 ± 0.9, respectively; *p* = 0.047 for the number of chronic illnesses and *p* < 0.0001 for the Charlson index). Finally, patients with dementia rated their health status at a lower level (6.0 ± 2.4) compared to MCI participants (6.5 ± 2.2) and non-impaired participants (6.4 ± 2.1; *p* = 0.051).

**Table 4:** Frequency of chronic illnesses in individuals diagnosed with dementia, MCI, and cognitively non-impaired participants.

**Chronic illness**	**Non-impaired** **(*n* = 146)**	**MCI** **(*n* = 231)**	**Dementia** **(*n* = 126)**	***P*-value**
Anemia	5 (3.4%)	13 (5.6%)	11 (8.7%)	0.172
Anxiety disorder	9 (6.2%)	13 (5.6%)	9 (7.1%)	0.851
Hypertension	99 (67.8%)	168 (72.7%)	79 (62.7%)	0.141
Arrhythmia	16 (11.0%)	26 (11.3%)	15 (11.9%)	0.969
Arthritis	14 (9.6%)	37 (16.0%)	11 (8.7%)	0.066
Benign prostate hyperplasia	16 (30.2%)	15 (23.8%)	14 (29.8%)	0.690
CHD	22 (15.1%)	27 (11.7%)	30 (23.8%)	0.011
COPD	15 (10.3%)	20 (8.7%)	14 (11.1%)	0.732
Dyslipidemia	70 (47.9%)	101 (43.7%)	51 (40.5%)	0.458
Depression	19 (13.1%)	39 (16.9%)	37 (29.4%)	0.002
GERD	24 (16.4%)	57 (24.7%)	21 (16.7%)	0.078
Glaucoma	14 (9.6%)	13 (5.6%)	9 (7.1%)	0.348
Hyperuricemia	14 (9.6%)	15 (6.5%)	7 (5.6%)	0.379
Hypothyroidism	22 (15.1%)	28 (12.1%)	9 (7.2%)	0.130
Peptic ulcer	16 (11.0%)	17 (7.4%)	13 (10.3%)	0.442
Osteoporosis	31 (21.4%)	69 (30.0%)	22 (17.5%)	0.019
Stroke	2 (1.4%)	4 (1.7%)	4 (3.2%)	0.529
Type-II diabetes	43 (29.5%)	56 (24.2%)	29 (23.0%)	0.406
Vertigo	10 (6.8%)	28 (12.1%)	14 (11.1%)	0.248
Number of chronic illnesses (mean, SD)	3.5 (2.1)	3.6 (1.9)	4.1 (1.9)	0.047
Charlson index (mean, SD)	4.2 (0.8)	4.4 (0.9)	4.9 (0.9)	<0.0001
Health status self-assessment (mean, SD)	6.7 (2.1)	6.5 (2.2)	6.0 (2.4)	0.0051

### Machine Learning Methods for MMSE Feature Selection

Participants were grouped as impaired (MCI or dementia; *n* = 357) vs. non-impaired *n* = 146. In Table 5 the results for the LASSO generalized linear model identified the following seven MMSE items as accounting for significant group differences: MMSE 5 (estimate −1.97; *p* = 0.040) MMSE13 (estimate −0.75; *p* = 0.015), MMSE19 (estimate −0.94; *p* = 0.021), MMSE20 (estimate −0.75; *p* = 0.021), MMSE22 (estimate −1.39; *p* < 0.0001), MMSE23 (estimate −0.72; *p* = 0.012) and MMSE26 (estimate −1.07; *p* < 0.0001). These items are listed in Table 6.

**Table 5:** Lasso generalized linear model for feature selection.

**MMSE items**	**Estimate**	**Standard error**	**T-statistic**	***P*-value**
MMSE5	−1.97	1.07	−1.84	0.040
MMSE13	−0.75	0.31	−2.42	0.015
MMSE19	−0.94	0.41	−2.29	0.021
MMSE20	−0.75	0.42	−1.80	0.030
MMSE22	−1.39	0.33	−4.18	<0.0001
MMSE23	−0.72	0.29	−2.49	0.012
MMSE26	−1.07	0.27	−3.94	<0.0001

*494 observations, 476 error degrees of freedom Dispersion: 1*.

*Chi^2^-statistic vs. constant model: 238, p = 7.21e-41*.

**Table 6:** Training, testing, validation and overall confusion matrices for the classification of participants as impaired (dementia/MCI) versus non-impaired.

	**Training**			**Testing**		
	**Number**	**Number**	**Correctly classified**	**Number**	**Number**	**Correctly classified**
Non-impaired	57	25	69.5%	17	4	81.0%
Impaired	45	227	83.5%	6	49	89.1%
Overall			80.2%			85.5%
	**Validation**			**Total**		
	**Number**	**Number**	**Correctly classified**	**Number**	**Number**	**Correctly classified**
Non-impaired	13	4	76.5%	87	33	72.5%
Impaired	7	52	88.1%	58	328	85.0%
Overall			85.5%			82.0%
**MMSE items following dimension reduction (item # in the Greek MMSE in parentheses)**						
1. (item 5)	What season is this?					
2. (item 13)	Repeat phrase					
3. (item 19)	Calculation (93 minus seven)					
4. (item 20)	Calculation (86 minus seven)					
5. (item 22)	What were the three objects (object 1) I asked you to remember?					
6. (item 23)	What were the three objects (object 2) I asked you to remember?					
7. (item 26)	Copy pentagon					

*Sum of squares error: Training 41.191; Testing 12.796. Epoch: 15 iterations, 6 validation checks, gradient 0.0069, best validation performance 0.43 at epoch 9*.

**Figure 1:**
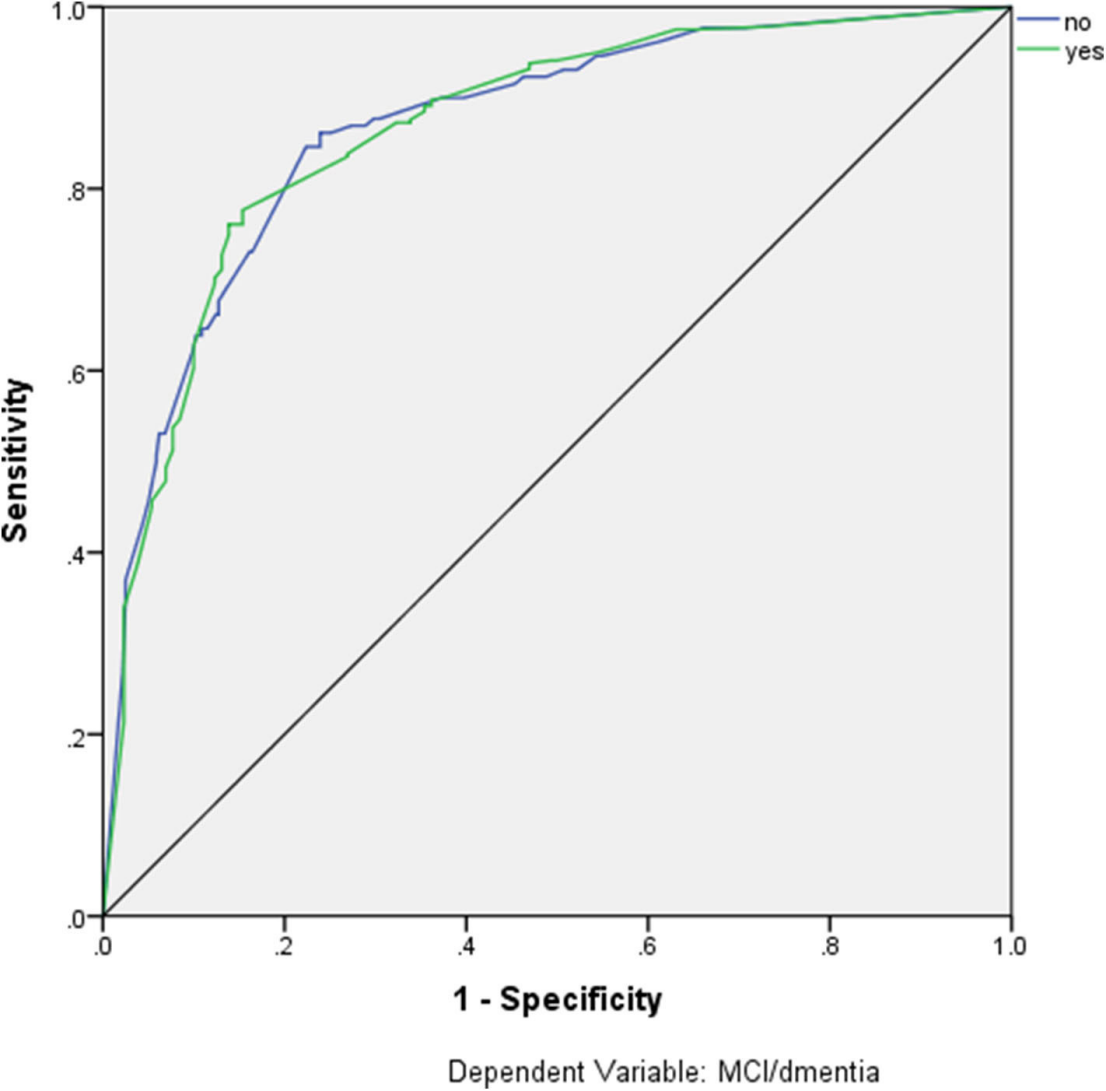
ROC Curve of the selected items in detecting dementia or MCI.

Artificial neural networks were used in order to estimate the diagnostic accuracy of these seven MMSE items for the classification of participants as impaired (MCI or dementia) and non-impaired. These seven items are listed in detail in Table 6, along with the confusion matrices for the training, testing, validation, and overall sample, revealing overall 85% Positive Predictive Value (PPV), 72.5% Negative Predictive Value (NPV), and overall 82.0% correct classification rate. The ROC curve for this model is depicted in Figure 1, with the area under the curve being 0.866.

## Discussion

### Summary of Main Findings

The present study indicated that a significant proportion of PHC visitors aged 60 years or older had low MMSE scores, with the prevalence of low MMSE scores being almost double in women compared to men. Most common chronic-illnesses were endocrine, nutritional, and metabolic diseases and the diseases of the circulatory system, with most chronic illnesses being more prevalent in women and a significant proportion of participants suffered from multi-morbidity. Logistic-regression models indicated that mental and behavioral disorders as well as diseases of the central nervous system were significant predictors of low MMSE-scores in both genders. Moreover, prevalence of several chronic illnesses, total number of chronic illnesses, and Charlson index scores were higher in patients formally diagnosed with dementias (major neurocognitive disorders) compared to patients with MCI (minor neurocognitive disorder) and individuals without neurocognitive disorder. Finally, machine learning algorithms indicated that seven out of the 30 total MMSE items could provide adequate classification rates of participants as cognitively impaired (suffering from MCI or dementia) and non-impaired.

### Results Under the Light of Literature

#### Mapping the Occurrence of Possible Cognitive Impairment

Our study indicated that one out of five participants aged 60 years or older had a low MMSE score indicating probable cognitive impairment. In Europe, reported rates of dementia range from a low 4.3% to a high 11.8% in those aged 65 years or older, with a notable scarcity of nationwide surveys (32). A previous study conducted in a primary care setting in Northern Greece indicated that 37.6% of men and 41.6% of women aged 65 or older showed various degrees of cognitive impairment (20). A recent random door-to-door study that was conducted in Central Greece reported a prevalence of dementia at 5.0% and a prevalence of MCI ta 7.4% in individuals aged 65 years or older (24–26). In our cohort, a previous study reported the prevalence of dementia at 10.8% and MCI at 32.4%, respectively, with the highest dementia rate (27.2%) reported in those aged 80–84 years old who also had the lowest level of education due to world war II (22). Our study highlighted a significant difference in the proportion of women with low MMSE scores compared to men. Besides the impact of gender, this difference could also be attributed among other potential factors to the lower levels of education and the higher prevalence of depression amongst participating women (33, 34). However, more evidence is needed for safe explanations.

#### Cognitive Impairment and Chronic-Illness Complexes Reported in PHC

In our study the presence of mental and behavioral disorders, diseases, and injury of the central nervous system, poisoning, and certain other consequences of external causes increased the odds of cognitive impairment (based on MMSE scores), while diseases of the musculoskeletal system and connective tissue reduced the odds in women. Adverse associations between cognition and depression, which is known to be the second most frequent neuropsychiatric symptom in patients with dementia (35), have been previously reported by our group and others (23, 36, 37). Moreover, a recent meta-analysis by Ford et al. used routinely collected primary care data in order to predict dementia (13). Findings of that study indicated that neuropsychiatric symptoms including depression, anxiety, seizures, and stroke were positively associated with dementia. A possible explanation for the associations between mental health disorders and cognition may be common neuropathological pathways through neuroinflammation (36). Furthermore, intracranial injury was also associated with all-cause dementia (OR 1.50; 95% CI 1.15–1.94) and rheumatoid arthritis [RA] was inversely related with all-cause dementia (OR 0.92; 95% CI from 0.83 to 1.02) (13) which could be due to the systematic use of non-steroid anti-inflammatory drugs of patients with RA (38). A claims data-based study with community-dwelling individuals in Germany also reported that individuals with dementia were more likely to be diagnosed with 15 co-morbidity complexes. Significant associations included mental and behavioral disorders (psychotic and neurotic disorders, depression, and insomnia) as well as diseases of the central nervous system like Parkinson's disease (39).

#### Cognitive Impairment and Multi-Morbidity

In the present study the crude number of chronic illness was not associated with cognitive impairment (based on MMSE scores). On the other hand, the Charlson index was associated with increased odds of cognitive impairment in men and marginally increased among participating women. The MRC CFAS cohort in the UK examined the percentage of medical co-morbidities in non-impaired, MCI, and patients with dementia and did not find any significant pattern difference between three groups (40). On the other hand, a nationwide survey in Taiwan reported a significant difference in the crude number of chronic illnesses between non-impaired and impaired (MCI and patients with dementia) individuals as well as a higher comorbidity index in the latter group (41). A recent large population-based study in the US concluded that the risk of MCI/dementia was elevated in persons with multi-morbidity (Hazard ratio 1.38; 95% CI 1.12–2.13). Finally, a retrospective population-based cohort study of all adults aged 65 years and older in Canada identified a higher median number of non-dementia morbidities between individuals with and without dementia suggesting that older age, multi-morbidity, and dementia are all strongly correlated (42).

#### Co-morbidities in Participants With Dementia, MCI, and Non-impaired Individuals

When comparing the frequencies of specific chronic illnesses between non-impaired individuals, individuals with MCI and individuals with dementia, we observed that the rates of CHD and depression were significantly higher in individuals with dementia and the rates of osteoporosis in individuals with MCI. The mean number of chronic illnesses and the Charlson index were significantly higher in persons diagnosed with dementia compared to those with MCI or non-impaired individuals. A retrospective longitudinal study from Taiwan compared baseline clinical characteristics of 279 patients and compared those who remained to MCI and those who progressed to dementia (43). They identified significantly higher extent of dyslipidaemia in those with stable MCI and a borderline higher rate of depression in those who progressed to dementia. Another large study from the same country compared diagnoses of 6,183 non-impaired individuals, 1,576 individuals with MCI and 697 with dementia and failed to detect significant differences in rates of specific chronic illness in pairwise comparisons of the three groups, although significant differences were identified in the mean number of comorbidities and the Charlson index (41). The MRC CFAS cohort in the UK compared health profiles between cognitively non-impaired with MCI and dementia patients and identified significantly higher prevalence of Parkinson's disease and stroke in the latter (40).

#### Machine Learning Toward a Brief Cognitive Test

The MMSE is one of the most widely used screening tests for dementia (27) briefly assessing a variety of cognitive domains (orientation, immediate memory, attention/concentration, delayed recall, language) (44). Several studies have worked on either improving its diagnostic accuracy or examined the performance of individual items or cognitive domains (45–47). On the other hand other shorter cognitive tests have been developed such as the Mini-Cog (48), the GPCoG (49) and Test Your Memory (TYM) (50, 51) since MMSE is considered lengthy and time-consuming (52). Furthermore, MMSE is not considered a sensitive test in detecting MCI (53). In our study, we grouped persons diagnosed with MCI or dementia together in comparison with cognitively non-impaired individuals. The seven items that provided adequate classification (impaired vs. non-impaired) rates were:

- What season is this? (orientation in time)
- Repeat phrase (repetition)
- Calculation (93 minus seven)
- Calculation (86 minus seven)
- What were the three objects I asked you to remember (episodic memory)
- Copy pentagon (visuo-constructive ability)

Various short forms of MMSE have been developed. Schultz-Larsen et al. used mixed-Rasch models and derived two subtests, each assessing distinct cognitive dimensions: orientation to time, attention/calculation, naming, repetition, and three-stage command (a-MMSE) and orientation to place, registration, recall, reading, and copying (B-MMSE) (54). In a subsequent study they concluded that a short form of MMSE was as accurate as the full version in predicting dementia (55). Another study developed a short version of MMSE based on the six memory items (three immediate recall and three delayed recall words) and have found a sensitivity for detecting dementia around 90% (56). Finally, the Montreal Cognitive Assessment (MoCA) screening tool was found to be highly sensitive (90%) in detecting MCI (57). The MoCa tool is assessing short-term memory, visuospatial abilities, executive functions, attention concentration and working memory, language and orientation to time and place. In our study, we used machine learning algorithms in order to develop a short form to detect dementia or MCI with most items being the same with the short forms discussed above. Although somewhat lower classification rates were achieved in the classification of either dementia or MCI that was somewhat expected since MMSE is not very sensitive in detecting mild forms of MCI (58).

### Strengths and Limitations

In this paper we combined results and measurements from the two phases of the CAC study: Phase I conducted in a community-based PHC setting conducted by GPs and PHC study nurses and Phase II conducted by secondary health care experts. To our knowledge, this project is the first large-scale study examining cognitive impairment in a primary health care setting in Greece. The Phase I sample size was large and used recruitment based on consecutive visitors rather than a door-to-door approach. This approach can provide a relatively accurate description of PHC visitors in a given region, yet its results may vary somewhat when compared to the general population. In the PHC arm of this study, the MMSE was used in order to detect cognitive impairment. We also used the education-adjusted cut-off points in order to improve specificity. Previously published data from this cohort of patients (utilizing DSM-IV criteria for diagnosis of dementia and MCI) indicated that 303 of 344 (88%) participants with MMSE scores <24 were diagnosed as having either MCI or dementia (22). So, we can be somewhat confident that the education-adjusted cut-off points represent individuals with either MCI or dementia. In our study we did not recruit participants visiting the selected PHC facilities for an emergency. To this end we probably have excluded cognitive impaired individuals due to delirium or other acute causes. Most of our recruited participants (~80%) visited the selected PHC facilities to renew prescriptions so most likely these individuals suffered from a chronic condition. Therefore, our sample may not include healthy older adults, as well as patients suffering from debilitating/life-threatening conditions that in Greece are typically treated within secondary health care/hospital settings. As regards the second sub-study of the project, although its limitations have been reported previously (22), it should be noted that the participation rates of those invited were modest which could impede generalization of the results, however no significant differences in basic demographics were found between those who agreed to participate and those who declined participation. Another limitation arises from the fact that all 30 MMSE questions were asked in the same order across participants. Our results indicated that seven of these items could comprise an abbreviated test for classification of patients as impaired or non-impaired. It is unclear whether a selective exclusion of specific items impedes the validity of the final test and it is a matter of future research to compare the validity of the abbreviated version vs. the full version of the MMSE. Furthermore, although our models indicated that the second and the third subtraction questions were more sensitive in detecting cognitively impaired individuals, it would probably make more sense to include the first subtraction question as well. We should note that this study was designed and implemented using the DSM-IV criteria and terminology. At the time of the study the DSM-V (1) criteria were published which introduced the terms of major and minor neurocognitive disorders as a replacement for the terms “dementia” and “MCI,” respectively, which are not formally included in this study. Finally, the cross-sectional nature of this project does not permit inferences regarding causal associations between chronic illnesses and dementia or MCI.

### Implication for Research, Education, and Clinical Practice

The findings of this study indicated high frequency of cognitive impairment which could indicate dementia or MCI in a primary care setting. Given the progressive nature of MCI and dementia in older individuals, screening protocols could be established within the context of a PHC consultation. General practitioners and PHC nurses and personnel could be trained to recognize early signs and symptoms and perform the appropriate diagnostic tests even in their short forms and also familiarize themselves with the updated terminology of the DSM-V criteria which aims to reduce the stigma associated with both the word dementia and the conditions that it refers to (1). Although no disease-modifying medication is currently available, early diagnosis would allow more time for those concerned to adjust while the patient can still actively engage in some activities (59). A timely diagnosis can also offer opportunities of early intervention, implementation of coordinated care plans, better management of symptoms, patient safety, cost savings, and postponement of institutionalization (60). Furthermore, GPs and PHC personnel should be trained in order to recognize and manage the associated co-morbidities. Specific illness-complexes, such as history of mental and behavioral disorders and diseases of the central nervous system, could serve as alarming signs for the presence of significant cognitive impairment. The nature and the complexity of the disease require a collaborative approach in the management of patients with dementia (61). A plethora of health care professionals could be involved in the process including dieticians (62), occupational therapists (63), speech therapists (64), music therapists (65), and others. In Greece, a recent health care reform took place with the introduction of integrated health care teams in urban primary care settings (66). These health care teams could be trained and serve as a collaborative approach in the identification and management of patients with cognitive impairment. Recent experience on other research topics indicated that intervention projects within the context of Greek primary care could be successfully implemented (67).

As regards future research, the findings of this study suggest that machine learning techniques could contribute to better, faster and simpler diagnostic procedures. Studies already published pointed out that machine learning techniques could help optimize algorithms to improve detection of dementia and/or progression from MCI to dementia based on health records. Machine learning techniques such as artificial neural networks have been applied in order to classify patients into dementia vs. non-dementia classes using structural, brain MRI scans. A recent study applied artificial intelligence techniques in the temporal analysis of spontaneous speech with promising results toward automatic screening for MCI in community settings (68). So, it is realistic to expect that a combination of simple question-based tests along with voice-pattern recognition will probably be able to classify correctly cognitive impairment.

## Conclusions

Cognitive impairment due to dementia or MCI in a community setting is a challenge for health-care services, clinicians, and the families of patients. This cross-sectional study from a Southern European setting provided new information about the extent and related co-morbidity of cognitive impairment due to dementia/MCI and suggested that the use of simplified instruments could be of valid use in the context of PHC consultations. Modern analytic tools such as machine learning could contribute to the development of faster and more accurate diagnostic procedures.

## Data Availability Statement

Data and materials for this study are available from the authors upon reasonable request. Due to restrictions stated in our ethical approvals data are not available on public data repositories.

## Ethics Statement

The studies involving human participants were reviewed and approved by the Bioethics Committee of the University Hospital of Heraklion (protocol number: 13541, 20.11.2010). The patients/participants provided their written informed consent to participate in this study. For patients unable to provide it, informed consent was provided by their caregivers.

## Author Contributions

AB: performed data entry, statistical analysis, and drafted the first version of the manuscript. CT, DB, CL, and AV: conceived the idea of the project. IZ: contributed to the project coordination and drafting a revision of the manuscript. ES, CT, SP, LM, IZ, MB, and PS: contributed to drafting and revision of the manuscript. AV: was the PI of the project and contributed to drafting and revision of the manuscript. CL: contributed to drafting and revision of the manuscript and was supervisor and coordinator of the PHC team. All authors have reviewed manuscript prior to submission.

## Conflict of Interest

The authors declare that the research was conducted in the absence of any commercial or financial relationships that could be construed as a potential conflict of interest.
